# Nortriptyline for pain in knee osteoarthritis: a double-blind randomised controlled trial in New Zealand general practice

**DOI:** 10.3399/BJGP.2020.0797

**Published:** 2021-06-02

**Authors:** Ben Hudson, Jonathan A Williman, Lisa K Stamp, John S Alchin, Gary J Hooper, Dee Mangin, Bronwyn F Lenox Thompson, Les Toop

**Affiliations:** Department of General Practice, University of Otago, Christchurch.; Biostatistics and Computational Biology Unit, University of Otago, Christchurch.; Department of Medicine, University of Otago; Canterbury District Health Board, Christchurch.; Pain Management Centre, Canterbury District Health Board, Christchurch, New Zealand.; Department of Orthopaedic Surgery and Musculoskeletal Medicine, University of Otago; Canterbury District Health Board, Christchurch.; Department of General Practice, University of Otago, Christchurch.; Department of Orthopaedic Surgery and Musculoskeletal Medicine, University of Otago, Christchurch.; Department of General Practice, University of Otago, Christchurch.

**Keywords:** analgesia, general practice, knee osteoarthritis, randomised controlled trial

## Abstract

**Background:**

Osteoarthritis (OA) of the knee is a common cause of chronic pain. Analgesics that are currently available have limited efficacy and may be poorly tolerated. Tricyclic antidepressants are used as analgesics for other chronic conditions, but they have not been evaluated as analgesics in OA.

**Aim:**

To investigate the analgesic efficacy of nortriptyline in people with knee OA.

**Design and setting:**

A two-arm, parallel-group, 1:1, double-blind, randomised, placebo-controlled trial in Christchurch, New Zealand.

**Method:**

Participants were recruited from orthopaedic outpatient clinics, primary care, and through public advertising. Adults with knee OA and a pain score of ≥20 points on the 50-point Western Ontario and McMaster University Osteoarthritis Index (WOMAC) pain subscale were randomised to receive either nortriptyline or identical placebo for 14 weeks. The primary outcome was knee pain at 14 weeks measured using the WOMAC pain subscale. Secondary outcomes included: function; stiffness; non-steroidal anti-inflammatory drug, opioid, and/or paracetamol use; each participant’s global assessment; and adverse effects at 14 weeks.

**Results:**

Of the 205 randomised participants, 201 (98.0%) completed follow-up at 14 weeks. The baseline-adjusted mean WOMAC pain subscale score at week 14 was 6.2 points lower (95% confidence interval = −0.26 to 12.6, *P* = 0.06) in the nortriptyline arm versus the placebo arm. Differences in secondary outcomes generally favoured the nortriptyline arm, but were small and unlikely to be clinically relevant. However, the following were all more commonly reported by participants taking nortriptyline than those taking a placebo: dry mouth (86.9% versus 51.0%, respectively, *P*<0.001), constipation (58.6% versus 30.4%, respectively, *P*<0.001), and sweating (31.3% versus 20.6%, respectively, *P* = 0.033).

**Conclusion:**

This study suggests nortriptyline does not significantly reduce pain in people with knee OA. The adverse effect profile was as expected.

## INTRODUCTION

Osteoarthritis (OA) is the most common joint disease and a major cause of pain, disability, reduced quality of life, and large healthcare costs;^1^^–^3 in addition, the burden of disease is predicted to rise.^4^^–^5 Osteoarthritis management is focused on advice, exercise, weight loss (if obese), analgesia, and maintenance of function.^6^ The analgesics currently recommended for OA are not ideal: paracetamol is minimally effective^7^ and has been linked with increased risk of mortality, cardiovascular disease, gastrointestinal bleeding, and renal adverse events;^8^ although oral non-steroidal anti-inflammatory drugs (NSAIDs) are effective in reducing pain,^9^ they are associated with renal, gastrointestinal, and cardiovascular toxicity.^10^ Opioids may also be used, despite poor evidence of efficacy, a significant side-effects burden, and risk of dependency.^11^^–^13 Patients may be offered intra-articular corticosteroid injections, but their analgesic efficacy and safety are uncertain.^14^ In addition, knee OA may be treated with joint replacement, but access to this intervention is limited by resource constraints in many countries^15^ and by patient comorbidity. For younger people (aged <60 years), delayed joint replacement may be desirable due to an increased lifetime risk of prosthetic failure.^16^ Given all of these factors, there is a need for more-effective and better-tolerated pain management for people with OA.

Central sensitisation may play an important role in the chronic pain of OA,^17^^–^18 and some centrally acting agents have been shown to reduce OA pain.^19^^–^20 Tricyclic antidepressants (TCAs) are used in other chronic pain conditions,^21^^–^22 but their analgesic effect in OA has not previously been evaluated; the aim of this study was to assess the efficacy and safety of the TCA nortriptyline for analgesia in knee OA when used in addition to participants’ usual analgesia.

## METHOD

### Trial design

A two-arm, parallel-group, 1:1, randomised, double-blind, placebo-controlled trial was conducted to investigate whether nortriptyline (25 mg–100 mg per day) provides clinically significant pain relief in patients with OA of the knee. A full protocol has previously been published^23^ and results are presented in accordance with the Consolidated Standards of Reporting Trials (CONSORT) guidelines and extension for harms.^24^^–^25

**Table:** How this fits in

Patients with knee osteoarthritis (OA) frequently require analgesics, but the analgesics commonly used are not ideal as they are either insufficiently effective or have serious side-effects. The authors hypothesised that tricyclic antidepressants (TCAs), which are used as analgesics for other chronically painful conditions, may be helpful for patients with OA. In this randomised, double-blind, placebo-controlled trial, it was found that one TCA, nortriptyline, did not significantly reduce pain or improve physical function, stiffness, or participants’ global assessment of the impact of their OA; as such, nortriptyline is unlikely to be a useful treatment for patients with knee OA.

### Participant recruitment and eligibility

Potential participants were largely recruited from urban and suburban areas of Christchurch, New Zealand’s second largest city. Invitation letters to participate were sent to people with knee OA who had been declined specialist orthopaedic assessment for knee replacement (by a referral triaging orthopaedic surgeon, who read the referral letters and examined accompanying prereferral X-rays) and returned to their GP for ongoing care. These people were identified on lists held by the Canterbury District Health Board orthopaedic department. The authors did not attempt further contact for those who did not respond to initial invitation letters.

A range of local marketing initiatives were used. These included advertisements and articles in local newspapers and bulletins; posters in primary healthcare premises, libraries, and clubs; and stalls in shopping centres. Research nurses provided individuals who responded to these invitations with study information by post or over the telephone, and conducted in-person screening assessments. For inclusion in the study, participants had to meet all of the following criteria:

- have primary knee OA defined according to the American College of Rheumatology clinical criteria for the classification of idiopathic OA of the knee;^26^
- have knee pain scoring ≥20 points on the Western Ontario and McMaster Universities Osteoarthritis Index (WOMAC) pain subscale (range: 0–50 points);^27^ and
- have been on a stable analgesic regime for, at least, the previous 2 months.

Exclusion criteria included:

- previous joint replacement of the study knee;
- intra-articular steroid injection in the previous 3 months;
- secondary OA;
- concurrent use of any antidepressant; and
- any established contraindications to TCAs.

The full list of exclusion criteria can be found elsewhere.^23^

### Randomisation and blinding

An unstratified, 1:1-allocation, computer-generated randomisation list with blocks of varying size (one to four) was prepared by the study statistician (https://cran.r-project.org/web/packages/blockrand/index.html). Participants, as well as all investigators and research staff who were enrolling participants, dispensing medication, and/or assessing outcomes, were blinded to the treatment allocation. To assess the effectiveness of blind allocation, participants and research nurses were asked which study arm they believed the participant had been allocated to at the final study visit.

### Trial regimen and procedures

Treatment occurred over 14 weeks and comprised an 8-week dose-adjustment phase and a 6-week steady-dose phase. Participants were instructed to commence taking one capsule (containing either nortriptyline 25 mg or placebo) daily for 2 weeks, at which time they were contacted by a research nurse by telephone who advised them to increase or decrease their dose by one capsule per day according to the participant’s level of knee pain and adverse effects. Further dose adjustments occurred at 4 weeks, 6 weeks, and 8 weeks, up to a maximum dose of four capsules daily (100 mg nortriptyline daily in the treatment group). At 8 weeks, participants were instructed to maintain their current dose until week 14, when the final assessment was undertaken. Throughout the study, participants were free to use and adjust their usual analgesic medication as prescribed by their GP, but were requested not to use any other antidepressants or receive intraarticular steroid injections.

### Outcome measures

#### Primary outcome

The primary outcome was self-reported pain in the affected knee at week 14 over the previous 48 hours, captured using the WOMAC 3.1 pain subscale.

#### Secondary outcomes

Secondary outcomes comprised:

- physical function, measured using the WOMAC pain subscale;
- stiffness, measured using the WOMAC pain subscale;
- global assessment, measured using a visual analogue scale (VAS);
- response to treatment, according to the Outcome Measures in Rheumatology and Osteoarthritis Research Society International (OMERACT–OARSI) responder criteria;^28^
- quality of life, using the RAND 36-Item Health Survey 1.0 (RAND 36);^29^
- the proportion of participants reporting adverse events and the severity of these events, measured using the Antidepressant Side-Effect Checklist at week 14; and
- use of NSAIDs and other analgesics.

WOMAC and VAS scores were standardised to a range of 0 (best-possible outcome) to 100 (worst-possible outcome). RAND 36 physical and mental component summary scores were calculated according to simple item sums.^30^

### Data collection

Information was collected at baseline by a research nurse in a face-to-face interview and included demographics, clinical characteristics, baseline measures of outcome variables (WOMAC 3.1 OA index, global assessment by VAS, and RAND 36), and analgesic use during the previous 2 weeks.

Interviews were conducted by a research nurse 14 weeks after commencing the study medication, and self-reported items were completed during these interviews. Interviews were conducted at the Department of General Practice, University of Otago, and lasted circa 20 min.

Adverse events were captured at weeks 2, 4, 6, 8, and 14 in free-text fields incorporated into each tool and coded by a research nurse and study investigator using the Common Terminology Criteria for Adverse Events, version 5.^31^ The occurrence and severity of expected adverse events resulting from antidepressant use at any time during the study were captured for all participants at week 14 using the Antidepressant Side-Effect Checklist.^32^ Unexpected adverse events were collected at any point of contact with the study team.

Analgesic use during the final 2 weeks of the study was recorded by participants in daily diaries. Daily doses were calculated for each analgesic, and pre-specified rules were used to derive equivalent doses of NSAIDs and opioids (see Supplementary Table S1).

### Sample-size calculation

The study was powered to detect the minimum clinically important difference for reduction in pain at 14 weeks, measured using the WOMAC pain subscale; this was deemed to be 10% of the scale maximum,^33^ with 90% power at a two-sided significance level of 0.05. It was calculated that a sample size of 200 was needed, which conservatively assumed a pooled standard deviation of 20 points, no correlation between baseline and follow-up scores, and an attrition rate of up to 15%.^34^

### Statistical analysis

The statistical analysis plan and analysis code were prepared by the study statistician and agreed on by the principal investigator following blinded review of the data but before the randomisation code was broken. Analysis was conducted using R (version 3.5.1). The primary analysis followed an intention-to-treat approach and missing data were imputed using multivariate normal multiple imputation.^35^ A *P*-value of <0.05 was considered to be statistically significant.

The mean absolute treatment effect in the primary outcome was estimated using linear regression modelling, with baseline WOMAC pain subscale scores included as a covariate. Similar analyses were conducted for the secondary outcomes (namely, physical function, stiffness, global assessment, and quality of life). Absolute and relative differences in the proportion of participants who responded to treatment, according to the OMERACT-OARSI responder criteria,^28^ were calculated with 95% confidence intervals (CIs) using generalised linear regression models.

Heterogeneity of treatment effects (subgroup) analysis followed the framework presented by Kent *et al*.^36^ Additional sensitivity analyses were performed adjusting for age, body mass index (BMI), sex, years with OA, use of assistive devices, presence of any chronic comorbidities, and mental health status.

Generalised mixed-effects hurdle models were used to estimate differences in:

- the daily likelihood of a participant using an analgesic; and
- the average number of ‘standardised’ analgesic tablets taken daily by those who took any.

A logistic model was used for the daily likelihood of a participant using an analgesic and a truncated generalised Poisson model was used for the average number of ‘standardised’ analgesic tablets taken daily by those who took any. Both models included time of observation, treatment group, and time by treatment as fixed effects, and subject as a random effect.

## RESULTS

### Participant recruitment and characteristics

**Figure 1.**
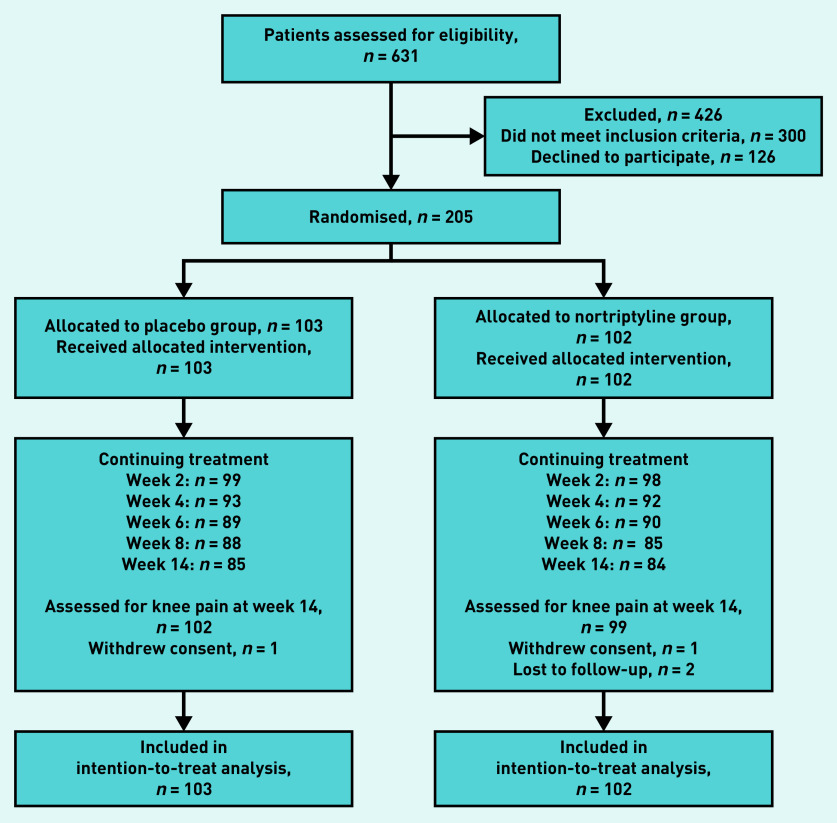
*Participant recruitment and randomisation. All participants were included at the final point of assessment irrespective of whether they had continued or ceased study medication.*

The participant recruitment process is outlined in Figure 1. A total of 205 participants were enrolled between November 2015 and October 2017, and the final follow-up interview was conducted in February 2018.

Participants’ demographic and clinical characteristics are presented in Table 1; their baseline characteristics were generally similar in both the placebo and nortriptyline arms, but the nortriptyline arm included a greater proportion of individuals who were obese (73.5% versus 47.6%), and the placebo arm a greater proportion of individuals who were overweight (46.6% versus 19.6%). Baseline VAS data were not collected for the first 24 participants (12 participants in both study arms) due to an error in the preparation of their record sheets. Multiple imputation assuming a multivariate normal distribution was used to impute the missing data for the baseline VAS at the same time that missing data was imputed for the outcome variables. The baseline VAS is relatively highly correlated with the WOMAC pain subscale and physical function scores (Pearson’s *r* = 0.4), which are also included as auxiliary variables in the multiple imputation models. Outcome data were not collected for four participants: two withdrew from the study after randomisation and two were lost to follow-up.

**Table 1.** Study participants’ characteristics at baseline in placebo (*n* = 103) and nortriptyline (*n* = 102) groups

**Characteristic**	**Placebo**	**Nortriptyline**
Mean age, years (SD)	64.6 (10.3)	64.4 (7.9)

Female, *n* (%)	43 (41.7)	44 (43.1)

**Ethnicity, *n* (%)^a^**		
European	87 (84.5)	96 (94.1)
Māori	9 (8.7)	12 (11.8)
Other	11 (10.7)	5 (4.9)

**BMI (kg/m^2^), mean (SD)**	31.3 (6.2)	33.2 (5.7)
Healthy: 18–<25, *n* (%)	6 (5.8)	7 (6.9)
Overweight: 25–<30, *n* (%)	48 (46.6)	20 (19.6)
Obese: ≥30, *n* (%)	49 (47.6)	75 (73.5)

Years with knee OA, mean (SD)	6.6 (7.1)	8.5 (7.9)

Use of assistive device, *n* (%)	39 (37.9)	41 (40.2)

Chronic conditions, *n* (%)	60 (58.3)	61 (59.8)

a *Ethnicity was self-identified; participants could select* >*1 ethnicity, so totals may add to* >*100%. BMI = body mass index. OA = osteoarthritis. SD = standard deviation.*

### Efficacy

#### Primary outcome

On average, participants given nortriptyline had a WOMAC pain subscale score at week 14 that was 6.2 points lower (95% CI = −0.26 to 12.56, *P* = 0.060) than that of those who received a placebo. Results were, effectively, unchanged in sensitivity analyses when adjusting for baseline covariates or excluding participants with protocol violations (Table 2).

**Table 2.** Primary and secondary continuous outcomes^a^ in placebo (*n* = 103) and intervention (*n* = 102) groups

	**Placebo, mean (SD)**			**Nortriptyline, mean (SD)**			**Baseline-adjusted difference at 14 weeks**		

	**Baseline**	**14 weeks**	**Change**	**Baseline**	**14 weeks**	**Change**	**Difference**	**95% CI**	***P-*value**
**Primary outcome**									
Pain (WOMAC)^b^	61.2 (12.5)	42.5 (24.0)	−18.7 (25.8)	60.2 (13.5)	36.0 (23.2)	−24.3 (22.5)	−6.2	−0.26 to 12.56	0.06

**Secondary outcomes**									
Function (WOMAC)^b^	59.9 (14.8)	41.9 (24.2)	−18.0 (23.2)	62.8 (15.0)	39.6 (24.4)	−23.2 (21.5)	−4.4	−10.48 to 1.79	0.16
Stiffness (WOMAC)^b^	61.2 (20.1)	47.2 (27.9)	−14.0 (29.5)	65.2 (20.3)	45.4 (27.4)	−19.9 (27.4)	−3.6	−10.94 to 3.72	0.33
Global assessment VAS^b^	72.6 (21.1)	53.6 (28.6)	−19.0 (33.0)	74.3 (20.2)	49.3 (30.8)	−25.0 (33.5)	−4.7	−12.91 to 3.46	0.26

**Quality of life^c^**									
Physical function	31.6 (9.9)	31.0 (10.1)	−0.6 (8.8)	29.9 (10.6)	31.9 (11.5)	2.0 (10.0)	2.0	−0.39 to 4.43	0.10
Role limitations due to physical health	39.7 (11.1)	39.7 (12.1)	−0.1 (11.5)	39.9 (11.0)	41.7 (11.9)	1.8 (11.9)	2.0	−0.95 to 4.88	0.19
Bodily pain	35.5 (7.5)	38.6 (8.8)	3.1 (9.4)	35.7 (8.1)	41.4 (10.0)	5.8 (8.8)	2.8	0.42 to 5.08	0.02
General health	46.1 (8.6)	46.7 (10.1)	0.6 (7.8)	47.4 (8.4)	47.4 (8.7)	0.1 (7.3)	−0.1	−2.15 to 1.88	0.90
Energy and vitality	47.1 (10.0)	47.1 (10.8)	0.0 (9.9)	46.2 (8.8)	46.8 (10.9)	0.6 (8.5)	0.3	−2.15 to 2.72	0.82
Social function	44.8 (11.4)	43.2 (13.5)	−1.6 (12.8)	44.7 (11.5)	45.0 (12.7)	0.4 (13.2)	1.9	−1.40 to 5.14	0.26
Role limitations due to emotional health	47.8 (11.4)	44.7 (12.9)	−3.1 (13.9)	47.3 (11.8)	46.0 (12.9)	−1.3 (11.8)	1.6	−1.63 to 4.79	0.33
Emotional wellbeing	52.1 (8.8)	51.7 (10.0)	−0.4 (8.9)	51.9 (9.1)	51.4 (10.9)	−0.6 (9.7)	−0.2	−2.66 to 2.21	0.86

a *All outcomes have been standardised to a range of 0–100.*

b *0 = best-possible outcome.*

c *100 = best-possible outcome. SD = standard deviation. VAS = visual analogue scale. WOMAC = McMaster Universities Osteoarthritis Index.*

#### Secondary outcomes

Participants in the nortriptyline arm achieved a greater improvement in the bodily pain subscale of the RAND 36 (baseline-adjusted difference 2.8, 95% CI = 0.42 to 5.08, *P* = 0.02) than the group receiving placebo; however, no statistically significant differences were observed in the other items of the RAND 36, the WOMAC subscales for physical function or stiffness, the VAS, or the proportion of responders (Table 2). There was no evidence of heterogeneity in treatment effects according to the predicted WOMAC pain subscore at week 14 (*P* = 0.82) or the predicted responder status (*P* = 0.67); as such, further subgroup analyses were not undertaken.

**Table 3.** Analgesic use^a^ by time in placebo (*n* = 99) and nortriptyline (*n* = 99) groups^b^

**Analgesic**	**Placebo**			**Nortriptyline**			**Adjusted odds ratio (95% CI)**
	**Baseline**	**14 weeks**	**Difference**	**Baseline**	**14 weeks**	**Difference**	
**Mean proportion of days when analgesic was not taken**							
Paracetamol, %	52.5	57.6	4.3	53.0	62.6	9.1	1.55 (1.03 to 2.37)
NSAID, %	66.6	69.1	4.1	61.6	73.9	11.5	3.91 (2.49 to 6.16)
Opioid, %	81.5	82.8	0.6	76.9	80.8	4.6	1.60 (0.86 to 2.99)
**Mean standard analgesic tablet count on days when analgesic was taken**							**Adjusted rate ratio (95% CI)**
Paracetamol, *n*	4.2	4.2	0.0	3.6	3.5	−0.1	0.97 (0.92 to 1.03)
NSAID, *n*	6.4	6.1	−0.3	7.4	6.7	−0.7	0.85 (0.80 to 0.90)
Opioid, *n*	7.0	6.6	−0.4	5.9	6.8	0.9	0.98 (0.90 to 1.07)

a *One pill is defined as the equivalent of 500 mg paracetamol, 200 mg ibuprofen, or 15 mg codeine.*

b *Of the 205 participants enrolled in the study (*n *= 103 placebo,* n *= 102 intervention), seven (*n *= 4 placebo,* n *= 3 intervention) did not complete a medication diary at week 14 and therefore could not be included in the analgesic analysis. Four withdrew or were lost to follow-up (*n *= 1 placebo,* n *= 3 intervention) and had no week 14 data, a further three (all placebo) completed the week 14 assessment (WOMAC questionnaire) but did not complete the medication diary. NSAID = non-steroidal anti-inflammatory drug.*

The estimated proportion of individuals who achieved the OMERACT-OARSI responder criteria was 56.4% (95% CI = 46.8 to 66.0) in those treated with placebo and 68.5% (95% CI = 59.4 to 77.6) in those treated with nortriptyline. This represents an absolute difference of 12.1% (95% CI = −1.4 to 25.6, *P* = 0.08) and a relative improvement of 1.2 (95% CI = 0.98 to 1.51, *P* = 0.08) (data not shown).

### Analgesic use

Participants in the nortriptyline group, in comparison with those receiving placebo, had a greater proportion of days when they did not take any NSAIDs (73.9% versus 69.1%, adjusted odds ratio [OR] 3.91, 95% CI = 2.49 to 6.16) or paracetamol (62.6% versus 57.6%, adjusted OR 1.55, 95% CI = 1.03 to 2.37). If they did take NSAIDs they took, on average, fewer tablets (adjusted rate ratio 0.85, 95% CI = 0.80 to 0.90) (Table 3).

### Adverse events

Seven serious adverse events (SAEs) were reported: one — for a participant receiving nortriptyline — was life threatening, and in both study arms three participants required hospitalisation (three for those in the placebo group, three for those receiving nortriptyline). Two SAEs (the hospitalisations for myocardial infarction and atrial fibrillation) were considered to be related to the study medication (Table 4).

**Table 4.** Adverse events^a^ (serious adverse events, overall, and most common) in placebo (*n* = 102) and nortriptyline (*n* = 99) groups^b^

**Event**	**Placebo, *n* (%)**	**Nortriptyline, *n* (%)**	***P*-value**
**Serious adverse events**			
Overall	3 (2.9)	4 (4.0)	0.72
Life-threatening myocardial infarction^c^	0 (0.0)	1 (1.0)	—
Hospitalisation for lower back pain	1 (1.0)	0 (0.0)	—
Hospitalisation for atrial fibrillation^c^	0 (0.0)	1 (1.0)	—
Hospitalisation for epistaxis	0 (0.0)	1 (1.0)	—
Hospitalisation for renal calculi	1 (1.0)	0 (0.0)	—
Hospitalisation for lung infection	1 (1.0)	0 (0.0)	—
Hospitalisation for hyperglycaemia	0 (0.0)	1 (1.0)	—

**Adverse events (Antidepressant Side-Effect Checklist at week 14)**			
Any adverse events	88 (86.3)	97 (98.0)	0.001
**Largest differences**			
Dry mouth	52 (51.0)	86 (86.9)	<0.001
Constipation	31 (30.4)	58 (58.6)	<0.001
Sweating	21 (20.6)	31 (31.3)	0.033
Sexual dysfunction	9 (8.8)	17 (17.2)	0.084
Headache	27 (26.5)	14 (14.1)	0.009
Diarrhoea	21 (20.6)	11 (11.1)	0.060

a *Each event is only recorded once per patient.*

b *Antidepressant Side-Effect Checklist data relates to those assessed for knee pain at week 14 only.*

c *Considered to be related to the study medication.*

For adverse events, dry mouth (86.9% versus 51.0%, *P*<0.001), constipation (58.6% versus 30.4%, *P*<0.001), and sweating (31.3% versus 20.6%, *P* = 0.033) were all more commonly reported by those taking nortriptyline, as was sexual dysfunction (17.2% versus 8.8%, *P* = 0.084), however, this was not statistically significant (Table 4).

### Test of blinding

When participants were asked what treatment they thought they received, the proportion who correctly guessed was similar among those who guessed that they were taking nortriptyline (62.7%, 95% CI = 53.3 to 71.4) and those who guessed that they were taking placebo (70.3% [95% CI = 58.5 to 80.3]) (*P* = 0.06) (data not shown).

## DISCUSSION

### Summary

In people with knee OA, adding nortriptyline to their usual analgesic treatment did not meaningfully improve pain, function, stiffness, or each participant’s global assessment of OA. Participants randomised to nortriptyline were more likely to have days when they did not take paracetamol or NSAIDs and, when they did take NSAIDs, they took fewer doses; however, these differences in analgesic use were small and unlikely to be clinically important. Dry mouth, constipation, sweating, and sexual dysfunction were more likely in participants taking nortriptyline, though sexual dysfunction was not statistically significant. Serious adverse events occurred in four participants taking nortriptyline and three taking placebo.

Of the SAEs, two were judged to be related to study medication and one was life-threatening (all of these occurred in participants taking nortriptlyine).

### Strengths and limitations

To the authors’ knowledge, this is the first published, double-blind randomised controlled trial of the analgesic effect of a TCA on OA. In addition, there are a number of strengths to the study design: primary and secondary outcome measures were recorded at 14 weeks, a follow-up period that is consistent with OMERACT-OARSI OA study recommendations;^37^ the primary outcome was measured using the WOMAC pain subscale, a valid, reliable, and responsive measure of pain and disability in OA;^38^^–^39 and the study was adequately powered to detect the minimum clinically important difference in pain. However, although this study was adequately powered to detect a clinically important effect of nortriptyline on pain, the sample size was too small to allow for subgroup analyses of participants who may have been predisposed to derive greater benefit from nortriptyline — for example, those with low mood or higher baseline levels of pain.

The pragmatic study design, in which study medication was taken in addition to usual treatment, is considered a strength: as a result of this, the effect of nortriptyline was tested in the context in which it would be used in clinical practice. However, as the design placed no limitation on participants’ use of other analgesics, it is possible that an analgesic effect of nortriptyline may have been masked that would have, otherwise, been apparent had the use of other analgesics been restricted.

Study participants were recruited from a number of sources. The majority were those with knee OA who had been declined assessment for knee replacement and returned to their GP’s care; others were recruited through a range of community-based advertising approaches. Participant demographics were broadly representative of patients with OA, though people of non-European ethnicity were under-represented. The recruitment of individuals referred for, and declined, specialist assessment may have meant that the range of disease severity in participants was greater than is typically encountered in primary care; it is important to be aware, therefore, that this may limit extrapolation of the findings to the full range of patients with knee OA that is encountered in primary care.

The study design also required participants’ study medication doses to be individually titrated according to their levels of pain and adverse effects believed by the participant to have been caused by study medication. This is important as nortriptyline metabolism and, hence, dosing have high inter-individual variability: the effective and tolerated daily dose ranges from <25 mg to >100 mg, so individualised dosing is essential.^40^^–^41 The choice of a dosing range from 25 mg to 100 mg daily could have been a limitation in that some participants may have received subtherapeutic dosing even at the maximum study dose, while others may have developed intolerable adverse effects at the minimum dose and ceased study medication as a result. However, the study dosing range was consistent with current clinical recommendations^40^ and was a pragmatic approach to permit flexible individualised dosing in the limited time available in the study. Furthermore, this process of dose adjustment closely resembles usual clinical practice when initiating a TCA.

The authors were able to demonstrate reasonably effective blinding of participants. This is of particular importance in trials of TCAs, as these medicines have a well-recognised set of adverse effects (in particular, dry mouth, constipation, and sedation) that may lead to unblinding, which could have compromised the internal validity of the study.

Participants’ baseline characteristics were generally similar in the placebo and nortriptyline arms. The nortriptyline arm, however, included a greater proportion of individuals who were obese. Individuals with a higher BMI report greater OA pain than those with lower BMI;^42^ therefore, the greater proportion of obese individuals in the nortriptyline arm may have biased results towards the null. However, mean BMI and the proportion of individuals with healthy BMI were similar in the two study arms, and the nortriptyline arm included a smaller proportion of those classified as overweight, so any effect on the findings is likely to have been small.

This may have introduced bias to the findings, although the mean BMI was similar in the two study arms, and adjusting for BMI and other key baseline variables did not substantially alter them.

### Comparison with existing literature

To the authors’ knowledge, this is the first trial of a TCA for pain in OA; therefore, no directly comparable studies exist. The null finding is disappointing as achieving adequate, well-tolerated, and safe analgesia for patients with OA is challenging, and few trials of analgesic agents demonstrate an effect size equal to the minimum clinically important difference.^11^^,^^19^^,^^33^

The evidence of TCAs’ efficacy in other chronic pain conditions is mixed: TCAs are established as first-line agents in neuropathic pain^43^ and have been shown to be effective in chronic headache,^22^ post-herpetic neuralgia, and diabetic neuropathy.^21^ Their efficacy in fibromyalgia is less clear,^44^^–^45 and a recent RCT of amitriptyline in chronic back pain did not show a statistically significant improvement in pain.^46^ In OA, the relative contribution of central and peripheral sensitisation and nociceptive pain varies between individuals;^47^ this may mean that some people are more likely to benefit from centrally acting analgesics than others.

### Implications for practice

Nortriptyline taken in addition to standard analgesia does not reduce pain in people with knee OA.

The degree of central sensitisation in an individual can be estimated using clinical scoring systems,^48^ and future work on centrally acting analgesics in OA could explore the potential for stratifying participants based on their degree of central sensitisation in order to determine whether centrally acting agents might usefully be targeted to those patients with higher levels of central sensitisation.
